# Changes in wrist joint contact area following radial shortening osteotomy for Kienböck’s disease

**DOI:** 10.1038/s41598-022-08027-0

**Published:** 2022-03-07

**Authors:** Junki Shiota, Daisuke Momma, Yuichiro Matsui, Nozomu Inoue, Eiji Kondo, Norimasa Iwasaki

**Affiliations:** 1 Department of Orthopaedic Surgery, Faculty of Medicine and Graduate School of Medicine, Hokkaido University, Sapporo, Japan; 2 Center for Sports Medicine, Hokkaido University Hospital, Kita 14, Nishi 5, Sapporo, Hokkaido 060-8638 Japan; 3 Department of Orthopedic Surgery, Rush University Medical Center, Chicago, USA

**Keywords:** Bone, Bone

## Abstract

We hypothesized that the contact area of the articular surface of the wrist joint could be evaluated using a custom-designed analytical program. The aim of the study was to compare the articular contact area of the wrist joint before and after radial shortening osteotomy for Kienböck’s disease. Nine wrists of 9 patients underwent radial shortening osteotomy for Kienböck’s disease. Computed tomography (CT) images of the wrist joint were reconstructed using a 3D reconstruction software package. Radioscaphoid and radiolunate joint contact areas and translation of the joint contact area from preoperative to postoperative were calculated using customized software. The mean Modified Mayo Wrist Score was significantly improved from 50.6 preoperatively to 83.3 at final follow-up (p < .001). Preoperatively, the pain was reported as severe in five wrists and moderate in four wrists, while at final follow-up, five patients were free from pain and four patients had mild pain with vigorous activity. The preoperative radioscaphoid joint contact area was 133.4 ± 49.5 mm^2^ and the postoperative radioscaphoid joint contact area was 156.4 ± 73.1 mm^2^. The preoperative radiolunate joint contact area was 194.8 ± 92.1 mm^2^ and the postoperative radiolunate joint contact area was 148.3 ± 97.9 mm^2^. The radial translation distance was 0.4 ± 1.2 mm, the dorsal translation distance was 0.6 ± 1.2 mm, and the proximal translation distance was 0.2 ± 0.4 mm. CT-based analysis revealed that the center of the contact area translated radially following radial shortening.

## Introduction

Kienböck’s disease refers to avascular necrosis of the lunate carpal bone, however no consensus is present regarding the primary causative factor of Kienböck’s disease. It is multifactorial, related to the following factors such as negative ulnar variance, local vascular abnormalities, and lunate morphology^1^. Clinical studies have indicated that excessive force on the lunate by the relatively longer radius leads to avascular necrosis of the bone, because of the positive correlation between the incidence of Kienböck’s disease and negative ulnar variance^2,3^. A number of studies have reported that radial shortening osteotomy provides acceptable clinical results for Kienböck’s disease^4^. However, because of the difficulty of obtaining direct measurements in vivo, few biomechanical studies have evaluated the articular contact area across the wrist joint following radial shortening.

Wrist joint contact patterns have been reported to reflect pathological conditions^5^. Kawanishi et al. reported that the wrist joint contact area of Kienböck’s disease was changed^6^. Based on this theory, Bey et al. reported that joint contact patterns are not only a more sensitive measurement than conventional kinematics for detecting subtle differences in joint function, but they may also provide a more clinically relevant indication of the extent to which a conservative approach or a surgical procedure has adequately restored normal joint function^7^. Therefore, the kinematics of the wrist joint in Kienböck’s disease before radial shortening osteotomy can be determined by measuring the wrist joint contact patterns. We hypothesized that the joint contact area of the wrist changes following radial shortening osteotomy. The aims of this study were to evaluate the contact area across the wrist joint in Kienböck’s disease, and to clarify the change in the contact area after radial shortening osteotomy.

## Methods

### Ethics statement

Our study was carried out in accordance with relevant guidelines of Hokkaido University Hospital and approved by the Research Ethics Review Committee of Hokkaido University Hospital. Our research protocols for human samples used in this study was approved by the Research Ethics Review Committee of Hokkaido University Hospital (approval ID:011-0327). Informed consents for the use of samples in our research were obtained from all participants.

### Patients

All patients underwent radial shortening between 1994 and 2019 for treatment of Kienböck’s disease. Computed tomography, Plane X-rays, and clinical assessment data were collected pre- and postoperatively. The Modified Mayo Wrist Scoring system was used for pre- and postoperative assessment of pain and wrist range of motion (ROM).

### Surgical technique

The surgical procedure has been described in detail previously^8,9^. The distal part of the radius was exposed between the brachioradialis and the flexor carpi radialis tendons. All surgical procedures were performed by two hand surgery specialists using previously described techniques^10^. Briefly, two parallel transverse cuts were made to remove a segment of bone (equal to the preoperative amount of positive ulnar variance) from the radius, followed by fixation at the osteotomy site by a 6-hole dynamic compression plate. Postoperatively, a short-arm orthosis was applied for 2 weeks, followed by active and passive motion.

### 3D bone model creation

CT images were obtained with a 320-slice multidetector 3D scanner with a wide field-of-view (FOV) (Aquilion One; Canon Medical Systems, Tochigi, Japan; slice thickness, 0.5 mm; slice interval, 0.5 mm; matrix 512 × 512; FOV φ500 mm). During image acquisition, the wrists were bandaged to carefully maintain a neutral position with the axes of the third metacarpal and forearm in neutral rotation, placed in a nonmetallic supporting frame. Axial CT images of the wrist were obtained before surgery and at 12 months postoperatively. Because a titanium fixation plate was used in all patients, there was no degradation of the CT image data due to metal artifact. CT images of each wrist joint were imported in DICOM format and segmented using a segmentation software package (Mimics 21R; Materialise, Leuven, Belgium). Three-dimensional images of the radius, ulna, scaphoid, and lunate were reconstructed, and the resulting 3D models were then exported as pointcloud and polygon models using the same software package. The 3D radius, ulna, scaphoid, and lunate bone models were then analyzed with custom-written software created using Microsoft Visual C +  + in the Microsoft Foundation Class programming environment (Microsoft, Redmond, WA) for further analysis^11–13^.

### Definition of joint contact area and anatomical coordinate system

**Figure 1:**
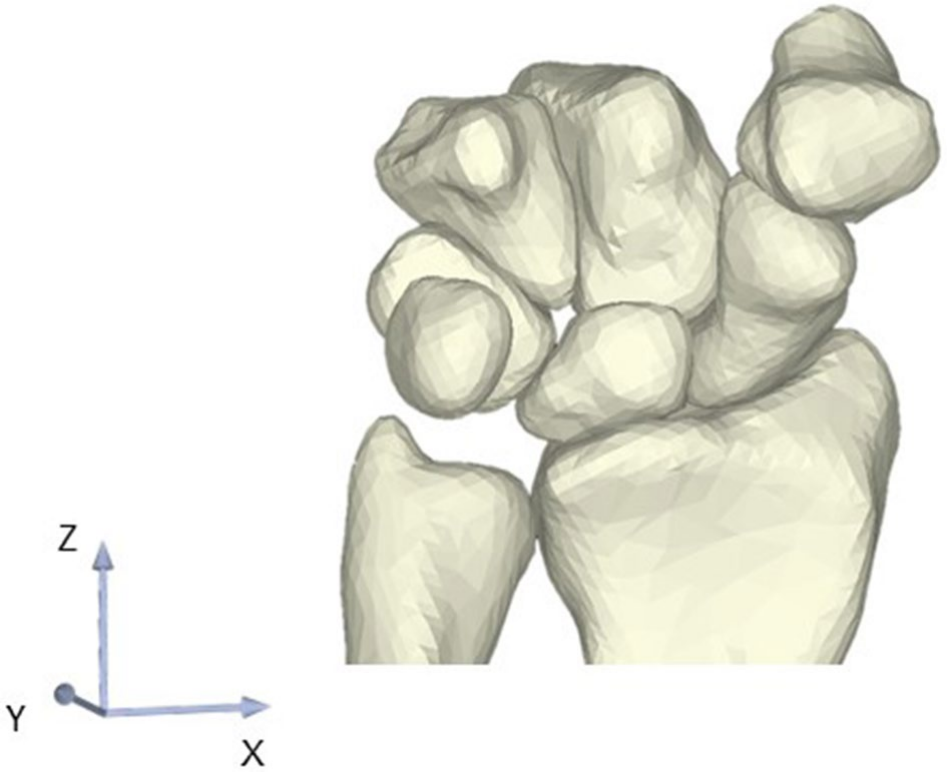
Anatomical coordinate system of the wrist. Translations along the X-, Y-, and Z-axes indicate the radial ( +)/ulnar (–), dorsal ( +)/volar (–), and distal ( +)/proximal (–) directions, respectively.

The surface-to-surface least-distance distributions between the radius and scaphoid models and between the radius and lunate models were calculated by a point-to-surface distance calculation algorithm using custom-written software^14,15^. Articular contact areas were defined as areas where the least distances were under a certain threshold level. The distance thresholds were determined with reference to previous studies of distances within the wrist joint space^16,17^. The threshold was 2.0 mm for each of the radioscaphoid and radiolunate joints. The radioscaphoid and radiolunate joint contact areas were calculated from the 3D bone models using custom-written software. The center of the contact area was also calculated, and preoperative to postoperative translation was also calculated using custom-written software. To evaluate translation of the center of the contact area, a validated 3D–3D registration method was used and a preoperative to postoperative transformation matrix was obtained^18,19^. We used the standard anatomical coordinate system for the wrist of the International Society of Biomechanics (Fig. 1)^20^.

### Statistical analyses

We conducted statistical comparisons between preoperative and postoperative Modified Mayo Wrist Scores and contact area using paired t-tests. We compared the translation of the center of the contact area between preoperative and postoperative using Wilcoxon signed-rank test. P values < 0.05 were considered significant. Data are presented as mean ± SD and the corresponding 95% confidence intervals.

## Results

### Participants’ demographics

Nine wrists of 9 patients that had been classified as Lichtman stage 2 (3 wrists), 3A (5 wrists), and 3B (1 wrist) underwent radial shortening osteotomy for Kienböck’s disease (Table 1). All patients reported a marked reduction in wrist pain at the 12-month follow-up. Preoperatively, the pain was reported as severe in five wrists and moderate in four wrists, while at final follow-up, five patients were free from pain and four patients had mild pain with vigorous activity. The average postoperative range of extension and flexion of the wrist (% of the normal side) increased from 70 ± 25% to 92 ± 8%. All patients except 2 were graded as excellent or good according to the Modified Mayo Wrist Scoring system (Table 1). The mean Modified Mayo Wrist Score was significantly improved from 50.6 preoperatively to 83.3 at final follow-up (p < 0.001) Plain x-rays of the wrist showed no apparent abnormal findings, including degenerative changes, except for the diseased lunate. Using the technique described by Gelberman et al.^21^ the mean preoperative ulnar variance on posteroanterior X-rays was 2.4 mm ulnar negative variance (range 1–4 mm ulnar negative variance) (Table 1). The postoperative value was 0.2 mm ulnar negative variance (range 1 mm ulnar positive variance to 1 mm ulnar negative variance) (Table 1).

**Table 1:** Participant characteristics.

Case	Sex	Age	Affected hand	Lichtman classification	Ulnar variance		Modified mayo wrist score	
					Pre-operation	Post-operation	Pre-operation	Post-operation
1	Male	39	L	II	− 3	0	45	60
2	Male	35	L	IIIa	− 2	0	70	90
3	Female	26	L	II	− 2	0	55	90
4	Male	21	L	IIIb	− 4	− 1	50	90
5	Male	44	L	IIIa	− 1	1	50	95
6	Male	22	R	IIIa	− 4	− 1	50	80
7	Female	55	L	IIIa	− 2	0	45	75
8	Male	34	R	IIIa	− 3	− 1	55	90
9	Male	43	L	II	− 2	0	35	80
Mean		35.4			− 2.4	− 0.2	50.6	83.3
p value pre- vs post-operation						< .001		< .001

### Joint contact area and translation of the center of joint contact area

**Figure 2:**
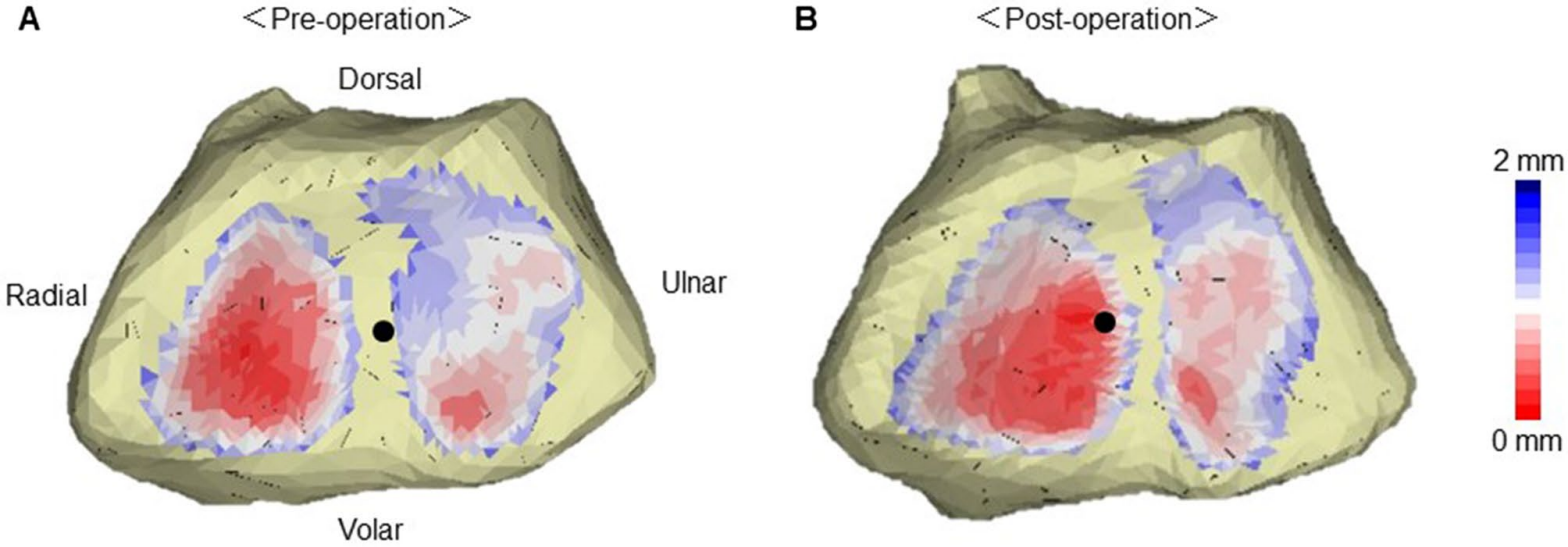
The representative bone models and joint contact area. (**A**) Preoperative and (**B**) postoperative wrist joint contact areas. Wide and narrow joint spaces are shown as blue and red areas, respectively. The black dot indicates the centroid of the joint contact area.

The preoperative radioscaphoid joint contact area was 133.4 ± 49.5 mm^2^ and the postoperative radioscaphoid joint contact area was 156.4 ± 73.1 mm^2^. The preoperative radiolunate joint contact area was 194.8 ± 92.1 mm^2^ and the postoperative radiolunate joint contact area was 148.3 ± 97.9 mm^2^. (Table 2, Fig. 2).

**Table 2:** Joint contact area.

Case	Contact area of scaphoid fossa, mm^2^		Contact area of lunate fossa, mm^2^	
	Pre-operation	Post-operation	Pre-operation	Post-operation
1	186.4	309.2	416	309
2	159.9	138.8	85.9	44.2
3	124.1	165.1	198.1	136.2
4	123	181.6	106.3	61.9
5	127.5	132.3	158.6	199
6	108.4	179.9	230.7	281.1
7	16.5	19.4	130.5	6.1
8	186.1	181.7	203.4	177.5
9	169.1	99.6	224	120.1
Mean	133.4	156.4	194.8	148.3
p value pre- vs post-operation		0.473		0.343

**Figure 3:**
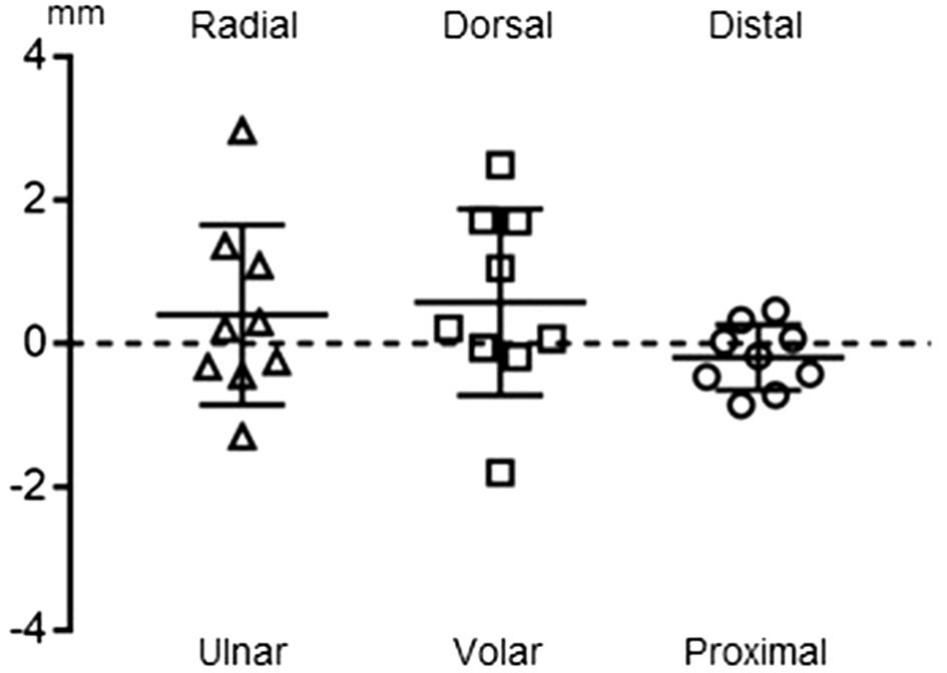
Postoperative translation of the centroid of the joint contact area.

When the postoperative translation of the centroid of the radioscaphoid and radiolunate contact area was decomposed into radial, dorsal, and distal directions, the radial translation distance was 0.4 ± 1.2 mm, the dorsal translation distance was 0.6 ± 1.2 mm, and the proximal translation distance was 0.2 ± 0.4 mm (Fig. 3).

## Discussion

To the best of our knowledge, this is the first in vivo evaluation of the contact area of the wrist joint before and after radial shortening osteotomy for Kienböck’s disease. The present study demonstrated that the contact area of the wrist joint changed following radial shortening osteotomy. The contact area of the center of the radioscaphoid and radiolunate joints was translated radially and dorsally following radial shortening osteotomy. This result indicates that radial shortening may change the load on the lunate by altering the contact area between the radiolunate and radioscaphoid joints.

Several cadaveric and theoretical studies have attempted to analyze the contact area of wrist joints in patients with Kienböck’s disease^22,23^. However, it is difficult for these biomechanical studies to create an experimental model simulating Kienböck’s disease and to determine the physiological loading conditions on the models. To overcome these difficulties, the current study applied an in vivo CT bone model analysis system to clarify alterations to the joint contact area after radial shortening osteotomy. In the present study, the contact areas of the radioscaphoid and radiolunate joints were calculated before and after radial shortening osteotomy using in vivo 3D methods. Using cadaveric wrist joints, Tencer et al. demonstrated that the overall scaphoid contact area was 1.47 times that of the lunate^24^, and Iwasaki et al. demonstrated that the mean value of the maximum area ratio of the lunate to the scaphoid fossa was 1.10 ± 0.44 (mean ± SD) in the normal wrist^25^. Padmore et al. reported that the radioscahoid joint contact area was 143.1 ± 40.3 mm^2^ and radiolunate joint area was 224.4 ± 75.9 mm^2^^26^. The current results of preoperative wrist joint contact area are comparable to those of previous reports, thus we revealed the wrist joint contact area after radial shortening.

A number of studies have reported that radial shortening provides acceptable clinical results for Kienböck’s disease^27–29^, and that this osteotomy achieves revascularization and unloads the diseased lunate^10,30,31^. Several biomechanical studies have demonstrated that radial shortening unloads the lunate by shifting the load toward the distal ulna^32,33^. Using a cadaveric model, Werner et al. showed that relative shortening of the radius by 2.5 mm shifted the load on the lunate from the radiolunate to the ulnolunate articulation^30^. Masear et al. and Trumble et al. used strain gauges mounted on the lunate to show unloading of the lunate following radial shortening or ulnar lengthening^32,33^. The present data showed that the contact area shifted radially and dorsally after radial shortening in living subjects, and all patients could return to their previous activities with no disturbance in wrist function. Therefore, it seems reasonable to conclude that decompression of the lunate is achieved by the biomechanical effects of radial shortening itself, in which the load on the lunate is shifted onto the scaphoid.

There are some limitations of the present study. First, we did not image the entire radius or evaluate the ulnar contact area. Second, we used a surface registration technique. Third, since the results varied greatly between patients, there may not have been a significant difference between the preoperative and postoperative joint contact area. Finally, the current study was not able to clarify direct relationships between wrist injury and various predictive factors. It will be necessary to conduct a prospective study with a larger number of participants in the future to show the relationship between contact area and wrist injuries; however, the present results consistently showed a characteristic pattern of contact area in the wrist that appeared to accurately represent the wrist contact area of patients with Kienböck’s disease, both before and after surgery.

In conclusion, bone surface modeling of the wrist joint using 3D CT imaging could evaluate the changes in the contact area after radial shortening osteotomy for Kienböck’s disease.
